# Oral health benefits of *Heyndrickxia coagulans*: a systematic review and meta-analysis of current evidence

**DOI:** 10.3389/froh.2025.1733955

**Published:** 2026-01-20

**Authors:** Silvia Cirio, Guglielmo Campus, Claudia Salerno, Aesha Allam, Maria Grazia Cagetti

**Affiliations:** 1 Department of Biomedical, Surgical and Dental Sciences, University of Milan, Milan, Italy; 2 Department of Cariology, Institute of Odontology, Sahlgrenska Academin, University of Gothenburg, Gothenburg, Sweden; 3 Department of Oral and Maxillofacial Sciences, Sapienza University of Rome, Roma, Italy; 4 Department of Cariology, Saveetha, Dental College and Hospitals, SIMATS, Chennai, India; 5 Department of Restorative, Preventive and Pediatric Dentistry, University of Bern, Bern, Switzerland

**Keywords:** dental caries, Heyndrickxia coagulans, meta-analysis, oral health, periodontitis, probiotics, systematic review

## Abstract

**Introduction:**

The oral microbiota plays a fundamental role in maintaining both oral and systemic health, while dysbiosis contributes to diseases such as dental caries and periodontitis. Probiotics have gained attention as adjunctive strategies to restore microbial homeostasis. *Heyndrickxia coagulans* (formerly *Bacillus coagulans*) is a spore-forming, lactic acid-producing bacterium with documented antimicrobial, antioxidant, and immunomodulatory properties. Its resilience to environmental stressors and industrial processing makes it a promising probiotic candidate. This systematic review and meta-analysis aimed to evaluate the effects of *H. coagulans* on oral health outcomes.

**Methods:**

A comprehensive search was performed across multiple databases up to September 2025 to identify randomized controlled trials (RCTs) and non-randomized studies assessing *H. coagulans* as a probiotic intervention for oral health. Data extraction followed PRISMA guidelines, and the risk of bias was evaluated using the RoB 2.0 and ROBINS-I tools. Meta-analysis was conducted using Stata SE® 18.5, with changes in salivary *Streptococcus mutans* levels as the primary outcome measure.

**Results:**

Eight studies (seven RCTs and one NRSI) met the inclusion criteria. Most were conducted in India, Iran, and North Macedonia, with sample sizes ranging from 30 to 183 participants aged 5–73 years. Administration of *H. coagulans*, via chewable tablets, mouthwash, or food matrices, resulted in a significant reduction of salivary *S. mutans* counts in both children and adults compared with placebo or other probiotics. Meta-analysis of four studies demonstrated a pooled effect size of −0.99 (95%CI = −1.60/0.39; *p* < 0.01), although substantial heterogeneity was observed (I² = 98.2%). Additional studies reported improvements in Gingival Index, bleeding on probing, and clinical attachment levels among participants with gingivitis or periodontitis. No significant adverse events were reported*.*

**Conclusion:**

*H. coagulans* appears to exert beneficial effects on oral health by reducing cariogenic bacterial load and improving periodontal parameters, supporting its potential use as an adjunct in caries prevention and gingival health maintenance. *H. coagulans* may favorably modulate the oral microbiota and contribute to overall oral health. However, further high-quality, large-scale clinical trials are needed to confirm these findings and define their therapeutic role in preventive oral care.

## Graphical Abstract

**Figure:**
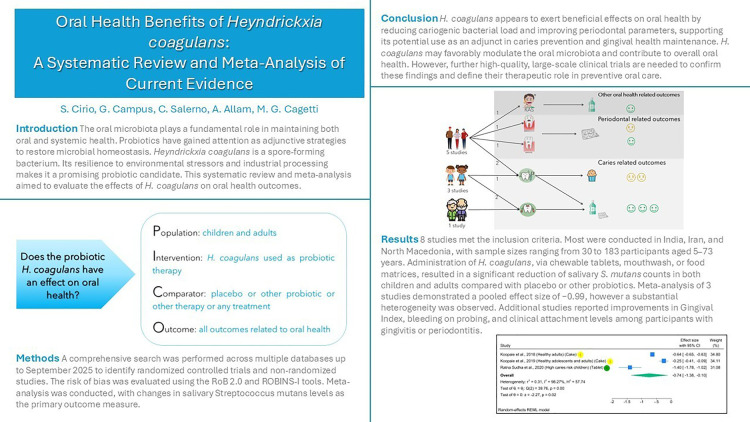

## Introduction

The major oral diseases, including dental caries and periodontitis, are associated with an imbalance in the oral microbiota (1). Alterations in its composition have been linked not only to oral diseases but also to systemic conditions such as diabetes, obesity, and cardiovascular disease (2, 3). Maintaining oral microbial homeostasis is increasingly recognized as a useful approach for promoting both oral and systemic health.

Probiotics have recently been proposed as an alternative approach to promote health. According to EFSA, probiotics must meet several criteria to be considered safe and effective: they must be non-pathogenic, lack transferable antibiotic resistance genes, be viable at the target site, adhere to and transiently colonize the mucosa, and demonstrate beneficial effects in well-designed clinical studies (4, 5). Probiotics exert their beneficial effects through multiple mechanisms, including competitive exclusion of pathogens, production of antimicrobial metabolites (organic acids, bacteriocins, hydrogen peroxide), modulation of the host immune system, and enhancement of epithelial barrier function (6). They can also help restore microbial balance after dysbiosis caused by antibiotics or infections.

Probiotics have shown potential benefits for oral health, including caries, halitosis, and periodontitis, in healthy individuals and those with systemic diseases (7–9). Some of the most studied and promising strains include *Lactobacillus rhamnosus, Lactobacillus reuteri, Lactobacillus acidophilus, Lactobacillus salivarius, Lactobacillus casei* and *paracasei, Bifidobacterium lactis*, among others (10). Collectively, these strains have demonstrated beneficial effects on the oral cavity. *Lacticaseibacillus rhamnosus* GG is one of the most extensively investigated strains and has shown the ability to counteract the most common oral diseases, including dental caries and periodontal disease (11–14).

Spore-forming probiotics are gaining popularity due to their enhanced survival and stability (15, 16). In functional food research, *Bacillus spp*. have attracted increasing attention due to their remarkable tolerance in the harsh conditions of the gastrointestinal tract. Furthermore, their superior stability during food and pharmaceutical processing and storage renders them ideal candidates for health-promoting formulations (17). In contrast, vegetative probiotic species are more sensitive to these processes and often require refrigeration to maintain potency (18).

*Heyndrickxia coagulans* (formerly *Bacillus coagulans*) is a Gram-positive, facultatively anaerobic bacterium belonging to the *Bacillus genus*, known for its ability to produce lactic acid (19). This strain exhibits antimicrobial, antioxidant, and immunomodulatory properties (20), and it has recently received considerable attention in dentistry (18, 21, 22). Recent studies highlight its effectiveness in controlling dental caries by reducing *Streptococcus mutans* and *Lactobacillus spp*. counts in plaque and saliva (18, 21). Additionally, they have been shown to lower gingival index scores, reduce bleeding on probing, and combat gingival inflammation (23). *H. coagulans* may exert these effects through several mechanisms: it produces antimicrobial metabolites, including organic acids and bacteriocin-like compounds that can inhibit cariogenic bacteria such as *S. mutans* (24, 25), and it can modulate host immune responses by enhancing anti-inflammatory cytokine activity and supporting mucosal immunity (26, 27). *H. coagulans* is listed by the EFSA under the Qualified Presumption of Safety status for recommended biological agents due to the absence of transferable antimicrobial resistance genes and the lack of toxigenic activity (28), unlike other *Bacillus spp* (29). This microorganism forms heat- and acid-resistant spores, enabling it to survive harsh environmental conditions (30). These features make *H. coagulans* particularly suitable for use as a probiotic in products subjected to industrial processing, including confectionery and functional foods (16).

## Aim

Given the increasing scientific interest in *Heyndrickxia coagulans* as a probiotic strain, recent studies have begun to explore its potential applications in the prevention and treatment of oral diseases. The aim of this systematic review is to summarize and critically assess the current evidence regarding the effects of *H. coagulans* on oral health.

## Materials and methods

### Protocol and registration

The present systematic review was registered *a priori* in the International Prospective Register of Systematic Reviews (PROSPERO) under protocol number CRD420251160000 (https://www.crd.york.ac.uk/PROSPERO/view/CRD420251160000) and was conducted and reported according to the Cochrane Handbook of Systematic Reviews of Interventions and to the guidelines of Preferred Reporting Items for Systematic Reviews and Meta-Analyses (PRISMA) statement (31, 32). The PRISMA checklists are displayed in Supplementary File - 1S*.*

### PICO question

This review sought to address the following question: “Does the probiotic *H. coagulans* have an effect on oral health?” To structure the clinical research question and establish the inclusion criteria (33), the PICO model was applied.

The PICO criteria were defined as follows:

- Population: children and adults.
- Intervention: *H. coagulans* used as probiotic therapy.
- Comparator: placebo or other probiotic or other therapy or no treatment.
- Outcome: all outcomes related to oral health.

### Eligibility criteria

Randomized clinical trials (RCTs) and non-randomized studies of interventions (NRSIs), including prospective, retrospective cohort studies, before-and-after comparisons, cross-sectional studies, case reports, case series, and non-clinical studies, were considered for inclusion. Articles had to focus on the use of *H. coagulans* to improve oral health. Studies for which full-text articles were not available were excluded.

### Information sources

Two authors (S.C. and A.A.) conducted an electronic literature search from inception to September 1, 2025. The search strategy included the keywords “*Bacillus coagulans,” “Weizmannia coagulans,” “Heyndrickxia coagulans,” “probiotics”* combined with terms related to oral health. Search strings were adapted for each database as detailed in Supplementary File 2S. All retrieved records were then imported and consolidated into the screening tool Ryyan® (34).

### Selection process

Following the removal of duplicate references, study selection was carried out independently and in duplicate by two reviewers. Titles and abstracts were screened, and articles not meeting the eligibility criteria were excluded. Full-text articles of potentially relevant studies were then retrieved and independently assessed by the same two reviewers. Any disagreements or uncertainties were resolved through discussion or, when necessary, by consultation with a third author (M.G.C.).

### Data collection process

Data extraction was conducted using a customized data collection form (Supplementary File 3S). The following information was recorded: authorship, year and country of publication, journal, study design, sample characteristics (including size, sex, and age), type of intervention and comparator (including dosage of probiotic, mode and timing of administration), and outcomes assessed. Numerical outcome data were extracted and, when possible, rounded to two decimal places; otherwise, data were reported as presented in the original source. Data on bacterial counts expressed in CFU/mL were converted to log₁₀ CFU/mL.

### Risk of bias in individual studies

The risk of bias was independently assessed in duplicate by two reviewers (S.C and C.S.), with any disagreements resolved through consultation with a third reviewer (G.C.), who provided the final judgment for each study. For RCTs, the revised Cochrane Risk of Bias tool (RoB 2.0) was applied. Responses to the signaling questions were entered into the Microsoft Excel® RoB 2 tool, which generated algorithm-based judgments for each domain as low risk, some concerns, or high risk. Visualizations of the results were produced using the Cochrane RoBvis web application (35).

For NRSIs, the ROBINS-I tool (Risk Of Bias In Non-randomized Studies of Interventions) was used (36).

### Summary measures and data synthesis

The sample size and the number of subjects were extracted for each study. To evaluate the effectiveness of the intervention, the difference between pre-treatment (mean ± SD_t0_) and post-treatment (mean ± SD_t1_) of the key variables was calculated. For each study, the mean change from baseline to final assessment was determined for both the treatment and control/placebo groups. The mean change in the treatment group (Δtest) was defined as the difference between the post-intervention mean and the baseline mean, and the same calculation was performed for the control/placebo group (Δplac). The treatment effect for each study was then expressed as the difference in mean changes between the two groups (Δdiff = Δtest−Δplac). The standard deviation (SD) of the change scores for each group was estimated using the baseline and final SDs, assuming a correlation coefficient of 0.5 between baseline and final measurements. The variance of the mean change for each group was obtained by dividing the squared SD of the change by the corresponding sample size. The standard error (SE) of the difference between groups was then calculated as the square root of the sum of these variances. A meta-analysis was conducted using Stata SE® 18.5 StataCorp LLC, StataCorp “meta command”, specifying Δdiff as the effect size and SE as its standard error. Forest plots were generated to visually display study-specific treatment effects with their 95% confidence intervals and the pooled overall estimate.

## Results

**Figure 1:**
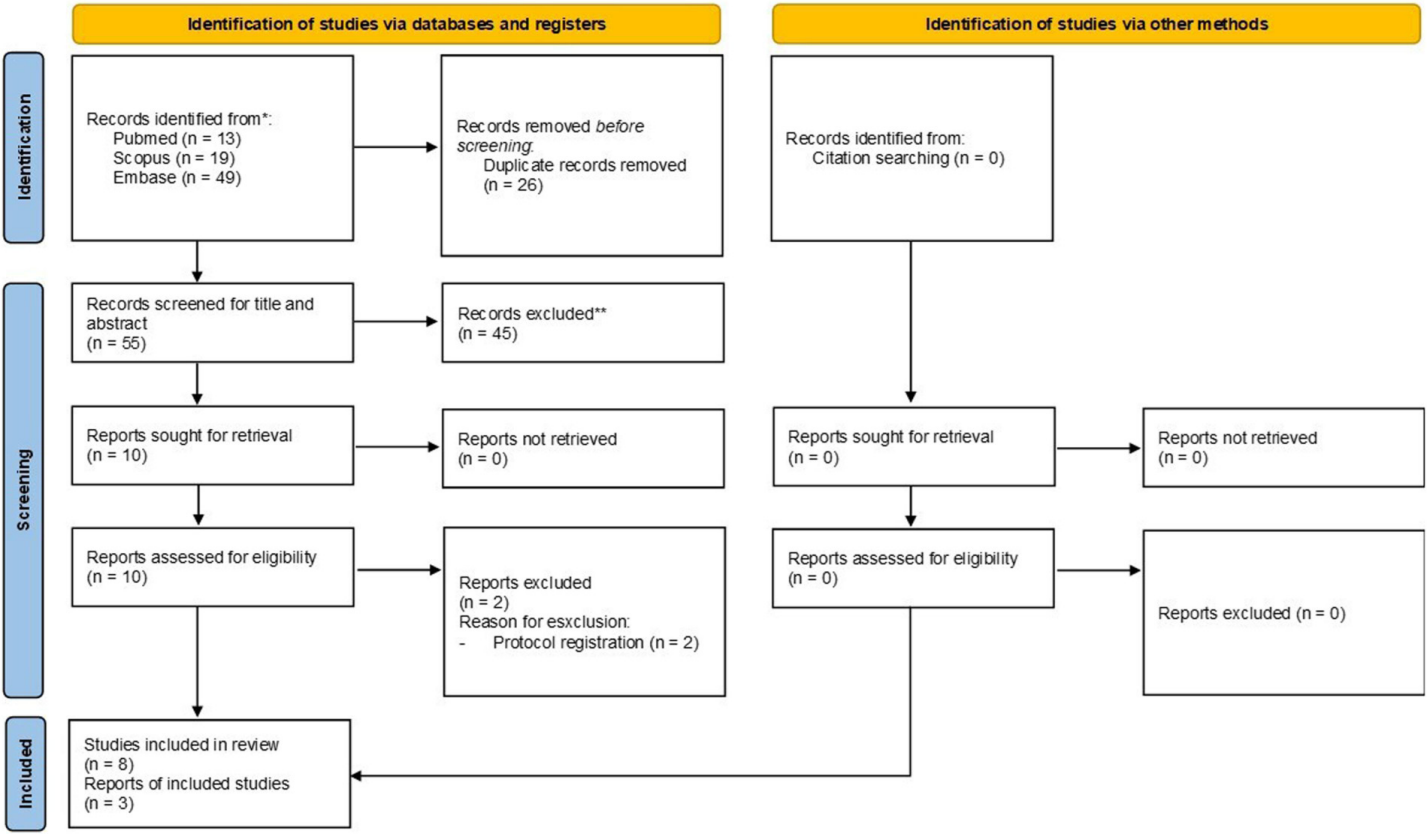
Prisma flow chart.

The results of the database search are illustrated in the flowchart in Figure 1. A total of 81 records were initially identified, and 55 remained after duplicate removal. Of these, 45 records were excluded by title and abstract screening (Supplementary File 4S). As a result, 10 articles were deemed eligible for full-text assessment (Supplementary File 5S). Two of these records (37, 38) were clinical trial registrations; therefore, a search was conducted to verify whether any articles reporting the results of these trials had been published, but none were found. Ultimately, eight studies (18, 21, 22, 39–43) were included in the qualitative synthesis, and three were included in the meta-analysis.

### Study types and geographic distribution

The majority of the included studies were conducted in India (18, 21, 39, 41, 43), two were carried out in Iran (22, 42), and one in North Macedonia (40). Seven studies were RCTs, and one was NRSI. All studies included were published between 2017 and 2022. Characteristics of the included studies are shown in Table 1.

**Table 1:** Main characteristics of the included studies.

Authors, years	Journal	Country	Type of study	Funding source
Jagadeesh et al., 2017 (23)	Nitte University Journal of Health Science	India	RCT	Not reported
Jindal et al., 2011 (21)	European archives of paediatric dentistry	India	RCT	Not reported
Koopaie et al., 2018 (42)	Journal of Babol University of Medical Sciences	Iran	RCT	Not reported
Koopaie et al., 2019 (22)	Dental and Medical Problems	Iran	RCT	International Campus of Tehran University of Medical Sciences (IC-TUMS), Iran
Krupa et al., 2022 (41)	Journal of Preventive Medicine and Hygiene	India	RCT	No funding received
Mitic et al., 2017 (40)	Research Journal of Pharmaceutical, Biological and Chemical Sciences	North Macedonia	NRSI	Not reported
Ratna Sudha et al., 2020 (18)	International Journal of Dentistry	India	RCT	Unique Biotech Limited, Hyderabad, India
Yendluru et al., 2020 (43)	European Journal of Molecular and Clinical Medicine	India	RCT	No funding received

### Sample sizes and age groups

Findings of the included studies are shown in Table 2. Sample sizes of the included studies ranged from 30 (40, 42, 43) to 183 subjects (74). Two studies included only pediatric participants (18, 21); one study included both children and elderly participants (41) and provided separate data for each age group. One study (42) included adolescents and adults, and four studies (22, 39, 40, 43) included only adults. The age of children ranged from 5 to 15 years, while the age of adults ranged from 15 to 73 years.

**Table 2:** Main findings of the included studies.

Author, year	Population	Sample (size, gender, age range)	Intervention	Outcomes	Arm	Baseline	Follow-up	p-Value intragroup	p-Value intergroup
Jindal et al., 2011 (21)	Healthy children	*N* = 150 (50/arm)	Mouthwash (*H. coagulans* SNZ 1969) 1.50*10^9^ CFU/day × 14 days (other probiotic treatment was *L. rhamnosus* + *Bifidobacterium* 1.25*10^9^ CFU/day)	Salivary MS (log_10_ CFU/mL)	Probiotic	Baseline vs. follow-up: 1.31 × 10^−7^		<0.001	Probiotic vs. placebo:<0.001; probiotic vs. other probiotic: NS
		M/F: 75/75			Other probiotic	Baseline vs. follow-up: 3.70 × 10^−5^		<0.001	
		Age range: 7–14 yy			Placebo	na	na	1.00	
Koopaie et al., 2018 (42)	Healthy adults	*N* = 30 (15/arm)	Cake (*H. coagulans*-specific strain and amount not reported) × 7 days	Salivary MS (CFU/mL)	Probiotic	7.87 (1.43) × 10^6^	4.65 (0.84) × 10^6^	NS	0.032
		M/F: 16/14			Placebo	7.87 (1.43) × 10^6^	21.39 (3.90) × 10^6^	0.021	
		Age range: 20–68 yy		Salivary pH	Probiotic	7.13 (0.56)	6.91 (0.44)	0.25	na
					Placebo	7.13 (0.56)	6.70 (0.64)	0.06	
Koopaie et al., 2019 (22)	Healthy adolescents and adults	*N* = 40 (x-over)	Cake (*H. coagulans*-specific strain and amount not reported) × 7 days	Salivary MS (CFU/mL)	Probiotic	6.42 (13.53) × 10^6^	6.95 (10.42) × 10^6^	NS	0.030
		M/F: 21/19			Placebo	6.42 (13.53) × 10^6^	1.23 (20.16) × 10^7^	0.027	
		Age range: 15–73 yy		Salivary pH	Probiotic	7.13 (0.49)	6.90 (0.23)	NS	na
					Placebo	7.13 (0.49)	7.00 (0.47)	NS	
Krupa et al., 2022 (41)	High caries risk children	*N* = 30 (10/arm)	Mouthwash (*L. acidophilus*-R 0052; *L. rhamnosus*-R 0011; *B. longum*-R 00175; ***B. coagulans*** SNZ 1969; *S. boulardii*) 1.50*10^9^ CFU/day × 14 days	Plaque MS (log_10_ CFU/mL)	Probiotic	6.88 (0.76)	4.97 (2.36)	0.023	na
		M/F: na			Chx	6.43 (1.28)	3.33 (2.71)	0.022	
		Age range: 5–12 yy			Xylitol	6.60 (0.96)	5.67 (1.12)	0.046	
	High caries risk elderly	*N* = 30 (10 per group)			Probiotic	7.16 (0.80)	5.55 (0.43)	0.018	
		M/F: na			Chx	7.13 (1.15)	4.91 (0.73)	0.004	
		Age range: >60 yy			Xylitol	6.42 (1.10)	5.03 (0.47)	0.009	
Ratna Sudha et al., 2020 (18)	High caries risk children	*N* = 48 (24/arm)	Chewable tablet (*H. coagulans* Unique IS2) 2.00*10^9^ CFU/day × 14 days	Plaque LB	Probiotic	2.71 (0.81)	1.54 (0.72)	<0.001	na
		M/F: 20/28		(log_10_ CFU/mL)	Placebo	2.72 (0.86)	3.50 (0.35)	<0.05	
		Age range: 5–15 yy		Salivary LB	Probiotic	3.70 (0.20)	2.94 (0.64)	<0.001	
				(log_10_ CFU/mL)	Placebo	3.30 (0.47)	3.83 (0.14)	<0.05	
				Plaque MS	Probiotic	2.33 (0.92)	1.82 (0.78)	<0.05	
				(log_10_ CFU/mL)	Placebo	2.71 (0.81)	2.56 (0.74)	NS	
				Salivary MS	Probiotic	3.86 (0.06)	2.56 (0.77)	<0.001	
				(log_10_ CFU/mL)	Placebo	3.08 (0.61)	3.18 (0.54)	NS	
				Plaque pH	Probiotic	6.00–6.30		NS	
					Placebo	6.00–6.30		NS	
				Salivary pH	Probiotic	7.60–7.80		NS	
					Placebo	7.60–7.80		NS	
Yendluru et al., 2020 (43)	Adults with RAS	*N* = 31 (17 in intervention/14 in comparison)	Mouthwash (*H. coagulans* SNZ 1969) 3.00*10^9^ CFU/day × 7 days	Ulceration size (cm)	Probiotic + TC_S_	1.24 (0.44)	4 days: 0.35 (0.49)		4 days: 0.110; 7 days: 0.008
		M/F: 15/25					7 days: 0.00 (0.00)		
		Age range: 18–50 yy			TC_S_	1.36 (0.50)	4 days: 0.64 (0.50)		
							7 days: 0.36 (0.50)		
				N of ulcerations	Probiotic + TC_S_	1.12 (0.33)	4 days: 0.53 (0.51)		4 days: 0.580; 7 days: 0.020
							7 days: 0.00 (0.00)		
					TC_S_	1.21 (0.43)	4 days: 0.43 (0.51)		
							7 days: 0.29 (0.47)		
				Pain (VAS)	Probiotic + TC_S_	2.53 (0.72)	4 days: 0.24 (0.56)		4 days: 0.004; 7 days: 1.000
							7 days: 0.00 (0.00)		
					TC_S_	2.57 (0.65)	4 days: 0.93 (0.73)		
							7 days: 0.00 (0.00)		
Jagadeesh et al., 2017 (23)	Adults with plaque-induced gingivitis	*N* = 30 (15/arm)	Chewable tablet (*H. coagulans*-specific strain not reported) 3.00*10^8^ CFU/day × 12 weeks	GI	Probiotic	1.60 (0.80)	1.50 (0.10)	*p* < 0.001	na
					Placebo	1.60 (0.2)	1.60 (0.30)	NS	
		M/F: na		PI	Probiotic	1.50 (0.60)	1.50 (0.20)	NS	
		Age range: 18–50 yy			Placebo	1.40 (0.80);	1.40 (0.70)	NS	
				BOP	Probiotic	81.30 (12.60)	75.89 (11.20)	*p* < 0.0001	
					Placebo	80.50 (20.80)	79.30 (21.20)	NS	
				GPx (pg/ml)	Probiotic	132.90 (21.90)	89.70 (15.50)	*p* < 0.0001	
					Placebo	131.00 (24.90)	131.60 (24.60)	NS	
Mitic et al., 2017 (40)	Adults with chronic periodontitis	*N* = 30 (15/arm)	Tablet (*H. coagulans*-specific strain not reported, *L. acidophilus, S. thermophilus, L. bulgaricus, B. bifidum*) 4.2*10^9^ CFU/day × 15 days	GI	SRP + probiotic	1.67	0.47	*p* < 0.001	NS
		M/F: na			SRP	1.67	0.47	*p* < 0.001	
		Age range: 18–50 yy		PI	SRP + probiotic	1.73	0.67	*p* < 0.001	NS
					SRP	1.53	0.33	*p* < 0.001	
				GBI	SRP+probiotic	1.33	0.27	*p* < 0.001	NS
					SRP	1.40	0.33	*p* = 0.006	
				PD (mm)	SRP + probiotic	4.93	3.97	*p* = 0.006	0.045
					SRP	5.00	4.73	*p* = 0.003	
				CAL (mm)	SRP + probiotic	4.37	4.20	*p* = 0.044	NS
					SRP	4.20	3.90	*p* = 0.17	

M, male; F, female; SD, standard deviation; N, numbers; MS, *mutans streptococci*; LB, lactobacilli; VAS, Visual Analogue Scale; GI, Gingival Index; PI, Plaque Index; BOP, Bleeding on Probing; GPx, Glutathione Peroxidase; GBI, Gingival Bleeding Index; PD, Probing Depth; CAL, Clinical Attachment Level; Chx, chlorhexidine; TC_S_, tetracycline; SRP, Scaling and Root Planing; NS, not significant.

### Caries-related outcomes

Five studies investigated the ability of *H. coagulans* to reduce the incidence of dental caries (18, 21, 22, 41, 42). In three studies, the population consisted of healthy subjects (21, 22, 42), two studies enrolled subjects at high risk of caries, one involving a pediatric population (18) and the other including both children and elderly participants (41).

In one study (41), *H. coagulans* was administered in combination with other probiotic strains, whereas in the remaining four studies, it was administered as a single strain (18, 21, 22, 42). In three studies, the daily dose ranged from 1.50 to 2.00 × 10⁹ CFU/mL, with an administration period of 14 days (18, 21, 41). In two studies, the administration period lasted 7 days; however, the daily dose was not reported (22, 42). In two studies, *H. coagulans* was administered through the consumption of a cake (22, 42); in two other studies (21, 41), subjects used a probiotic mouthwash, and finally, in one study, the probiotic was delivered via chewable tablets (18). In three studies, the comparator was a placebo (18, 22, 42); in one study, the probiotic was compared with a placebo and another strain (21); while in another study, *H. coagulans* was compared with chlorhexidine and xylitol (41).

Three studies investigated the effect of *H. coagulans* on salivary *Streptococcus mutans* (MS) levels (21, 22, 42), one assessed MS in dental plaque (41), and one evaluated both (18). In healthy adults, salivary MS counts increased significantly in the placebo group after the intervention period involving cake consumption, whereas no significant change was observed in the group receiving the probiotic (22, 42). In the study by Koopaie *et al*. (22), the difference between the two groups was statistically significant at 7-day follow-up (*p* = 0.03). Among children, a significant reduction in salivary MS counts was reported in the intervention group but not in the placebo group, both in healthy participants (21) and in those at high caries risk (18). One study compared two different probiotic mouthrinses (*H. coagulans vs. L. rhamnosus* + *Bifidobacterium*) and found a significant reduction in salivary MS in both groups (21). At the 14-day follow-up, the reduction in salivary MS levels was significantly greater in the *H. coagulans* group compared to the placebo group (*p* < 0.001); however, no significant difference was observed between the two probiotic groups.

Regarding MS in dental plaque, one study conducted on high caries risk children (18) reported a significant reduction in plaque MS in the probiotic group, but not in the placebo group. In the same study was also evaluated the effect of *H. coagulans* on *Lactobacillus* spp. (LB) counts (18). Results showed a significant reduction of salivary and plaque LB in the probiotic group, whereas a significant increase was observed in the placebo group. In another study, *H. coagulans* was administered as part of a probiotic mouthrinse formulation (*L. acidophilus*-R0052, *L. rhamnosus-*R0011, *B. longum*-R0175, *B. coagulans*-SNZ1969, *S. boulardii*) and compared with two alternative treatments, chlorhexidine-based mouthrinse and xylitol-based mouthrinse (41). All three interventions resulted in a significant reduction of plaque MS, with a greater effect observed in the elderly compared to children.

Finally, two studies (22, 42) assessed the effect of *H. coagulans* on salivary pH, while one study (18) evaluated both salivary and plaque pH. None of the studies reported significant changes in either the probiotic or the placebo group.

### Periodontal-related outcomes

One study evaluated the use of *H. coagulans* in adult patients with plaque-induced gingivitis (43). The probiotic was administered via mouthwash on 3.00 × 10⁸ CFU/mL daily dose. After three months of treatment, the Gingival Index (GI), Bleeding on Probing (BOP), and Glutathione Peroxidase (GPx) levels were significantly reduced in the probiotic group (*p* < 0.01), whereas no significant changes were observed in the placebo group. No significant differences in the Plaque Index (PI) were detected in both groups.

One study investigated the effect of *H. coagulans* in a group of adult subjects with chronic periodontitis (40). The probiotic was administered in tablet form in combination with other probiotic strains (*H. coagulans, Lactobacillus acidophilus, Streptococcus thermophilus, Lactobacillus bulgaricus,* and *Bifidobacterium bifidum*) at a daily dose of 4.2 × 10⁹ CFU, following scaling and root planing (SRP). The control group received SRP alone. At two-week follow-up, both groups showed significant improvements in GI, PI, Gingival Bleeding Index (GBI), and Probing Depth (PD). However, intergroup comparisons revealed a statistically significant difference only in GBI, in favor of the probiotic group (*p* = 0.04). Clinical Attachment Level (CAL) decreased in the control group, whereas it significantly increased in the probiotic group (*p* = 0.04).

### Other oral-related outcomes

Yendluru et al., 2022 investigated the effect of *H. coagulans* in combination with tetracyclines on ulcerative lesions in adult patients with Recurrent Aphthous Stomatitis (RAS) (39). The probiotic was administered via mouthwash at a dose of 2.00*10⁹ CFU/mL/day. The control group received tetracyclines alone. After 4 days of treatment, no significant differences were observed in the number or size of lesions between groups; however, the probiotic group reported significantly lower mean pain scores on the Visual Analog Scale (VAS) compared to the control group (*p* < 0.01). After 1 week, the probiotic group exhibited a significant reduction in both lesion size (*p* ≤ 0.01) and number (*p* = 0.02) compared to the control group, whereas no significant differences in pain scores were noted.

### Risk of bias assessment

**Figure 2:**
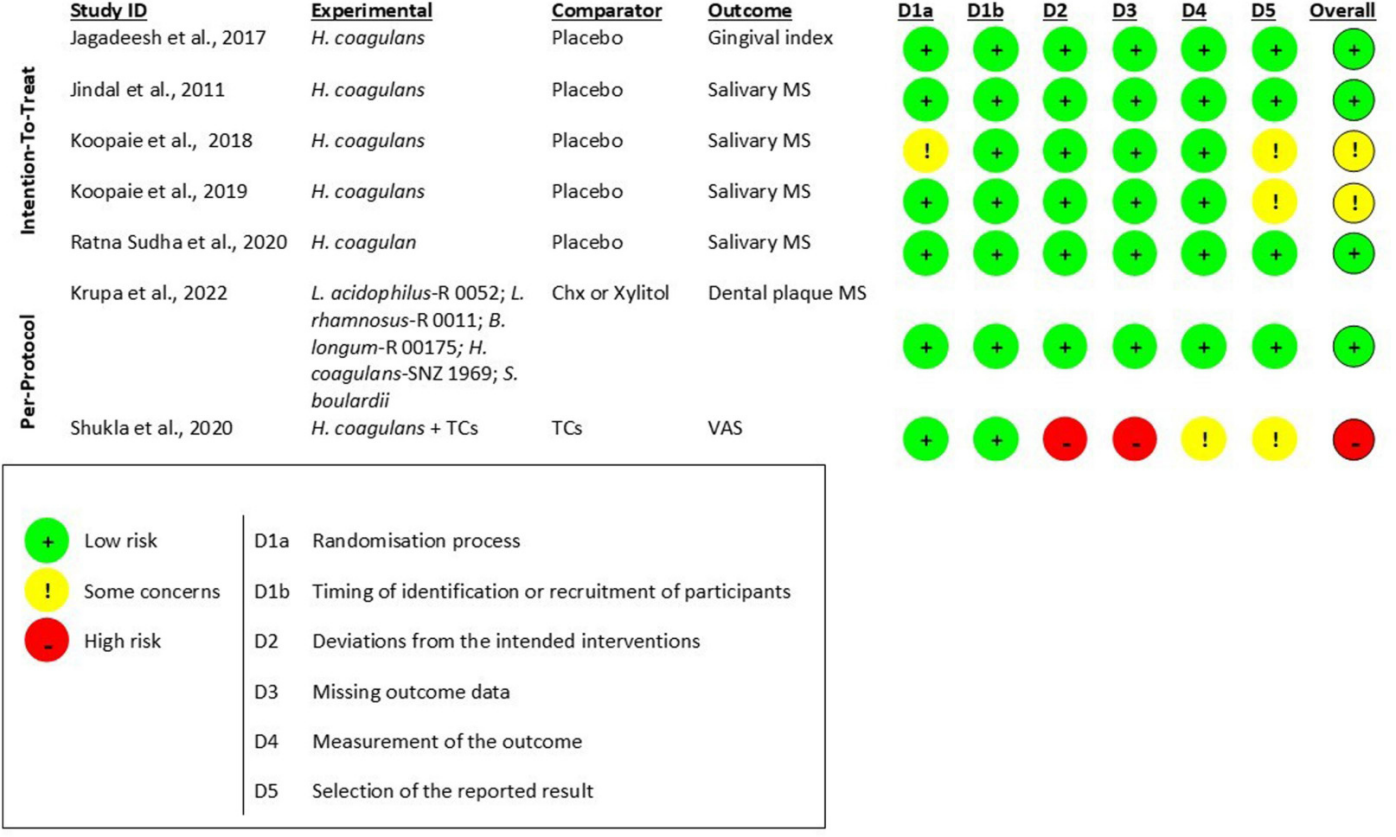
Risk of bias assessment of RCTs (RoB 2.0).

Seven studies (18, 21, 22, 39, 41–43) were assessed with the RoB 2.0 tool (Figure 2). Five studies (18, 21, 22, 42, 43) that did not explicitly report the use of an intention-to-treat approach were nonetheless analyzed using the tool for intention-to-treat studies, as they reported no dropouts. Two studies (39, 41) were classified as per-protocol. Overall, four studies (18, 21, 41, 43) showed low risk of bias, two (22, 42) raised some concerns, and one (39) had high risk of bias, mainly related to the selection of reported results due to the absence of protocol pre-registration and clear specification of planned analyses. One additional study (40), evaluated with ROBINS-I, was judged to raise some concerns (Supplementary File 6S). Assessments of publication bias (e.g., funnel plot inspection or Egger's test) were not conducted because the number of included studies was too small to provide reliable or interpretable results. Nevertheless, the comprehensive and systematic search across multiple databases reduces, although does not entirely eliminate, the likelihood of publication bias.

### Meta-analysis

**Figure 3:**
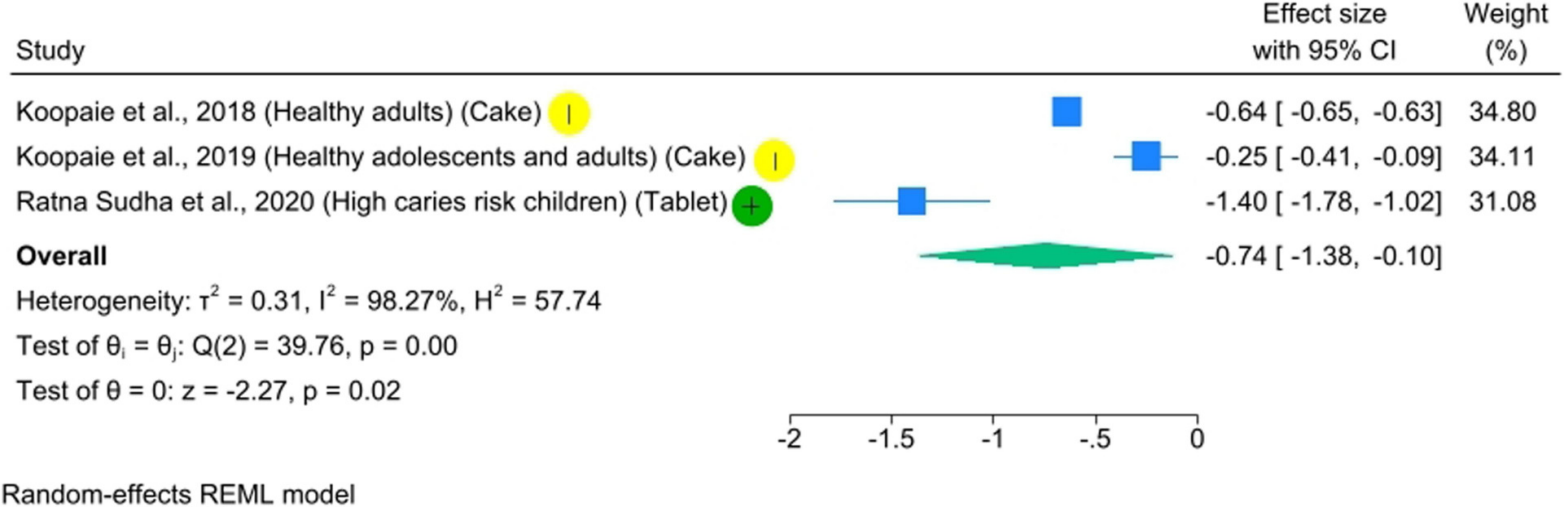
Forest plot for salivary mutans streptococci levels in children and adults.

Three studies were included in the meta-analysis (Figure 3), which represented the only quantitative synthesis feasible based on the available data. The outcome investigated was the reduction of salivary *S. mutans*. Individual study effect sizes ranged from −0.25 (95% CI: −0.41 to −0.09) to −1.40 (95% CI: −1.78 to −1.02), all favoring the probiotic intervention over control.

Although the pooled analysis showed a statistically significant reduction in salivary *S. mutans* levels (effect size −0.74, 95% CI −1.38 to −0.10; *p* = 0.02), the extremely high heterogeneity [*τ*² = 0.31; I² = 98.27%; H² = 57.74; Q(2) = 39.76, *p* < 0.001] considerably limits confidence in this estimate. Despite the fact that all included studies reported a reduction in *S. mutans*, the magnitude of this effect varied widely across studies. Therefore, the apparent consistency in the direction of the effect should not be interpreted as consistency in its strength or reliability.

## Discussion

This systematic review analyzed the available literature on the effects of *Heyndrickxia coagulans* (previously referred to as *Bacillus coagulans*) on oral health outcomes. Across seven included studies, *H. coagulans* demonstrated promising benefits, most notably in reducing salivary *Streptococcus mutans* levels and improving selected periodontal parameters. These findings suggest that *H. coagulans* may have a potential role as an adjunctive strategy for caries prevention and the management of gingival inflammation. However, these conclusions should be interpreted with caution due to the limited number of studies available, as well as their heterogeneity and methodological limitations.

Overall, the quality of the evidence is low to moderate. This assessment reflects several recurring methodological limitations across the included studies. In particular, the small number of studies and their limited sample sizes lead to substantial imprecision and reduce the robustness of the estimates. Considerable heterogeneity in populations, interventions, and outcome measures further compromises the consistency of the evidence. Moreover, the short intervention and follow-up periods do not allow for an adequate evaluation of long-term effects. Consequently, although the findings are promising, they should be interpreted with caution and confirmed by future, more rigorous research.

The most consistent finding across the included trials was a significant reduction in *Streptococcus mutans* counts following *H. coagulans* administration, particularly among children and individuals at high risk of caries. The high heterogeneity observed in the meta-analysis may be explained by several factors related to differences among the included studies. First, the meta-analysis is based on only three studies, comprising a total sample of approximately one hundred participants. The age of participants varied considerably: two studies (22, 42) involved adolescents and adults without providing stratified data that would allow age-specific subgroup comparisons, while one study focused on children (18). Additionally, one study included patients at high caries risk (18), whereas the other two (22, 42) involved healthy individuals, which may have amplified the observed effect in the high-risk group. Another important source of variability is the form in which the probiotic was administered. In two studies (22, 42), the probiotic was incorporated into a sweet food (cake), while in the third study (18) it was delivered through sugar-free chewable tablets. The consumption of cake, being a sugary food, led to an increase in *S. mutans* counts overall; however, this increase was less pronounced in the probiotic group, indicating a relative effect. In contrast, the chewable tablets did not contain sugar, resulting in an absolute reduction in *S. mutans* counts. Differences in probiotic dosage may also have contributed to heterogeneity, as two studies (22, 42) did not report the administered dose. Finally, although follow-up periods varied across studies, they were all relatively short, which likely had only a moderate influence on heterogeneity. Given these limitations, any subgroup or sensitivity analysis would be underpowered, potentially misleading, and unlikely to yield interpretable or clinically meaningful conclusions.

The effects on *Lactobacillus* spp. were less conclusive, with only one study reporting significant reductions. Considering that *Lactobacilli spp*. is primarily involved in the progression of dentinal caries rather than the initiation of the caries process, this partial effect may still hold clinical relevance; however, further confirmation in larger, well-designed trials is warranted. Nevertheless, the present results support the hypothesis that *H. coagulans* can meaningfully modulate the cariogenic microflora.

The impact of *H. coagulans* on plaque accumulation and gingival inflammation was variable. Some studies reported stabilization or reduction of plaque indices compared with control/placebo groups, while others found no difference. Notably, in gingivitis and periodontitis, *H. coagulans* was associated with improvements in the gingival index, bleeding on probing, and clinical attachment level. These results are encouraging and suggest a possible role in modulating host inflammatory response.

The findings of this review are in line with broader probiotic research, which has reported reductions in *S. mutans* levels in saliva and plaque (44, 45). Nonetheless, the evidence regarding effects on Lactobacilli and on clinical outcomes, such as plaque and gingival indices, remains inconsistent (46, 47). Although several meta-analyses have suggested modest improvements in gingival inflammation with probiotic use, these conclusions are tempered by high heterogeneity and generally low certainty of evidence (48, 49). Within this context, the present review provides preliminary insights into the potential role of *H. coagulans*, a species that may offer specific advantages, such as spore formation that could support survival and colonization. However, given the variability and limitations in the current evidence base, any species-specific claims should be interpreted cautiously until confirmed by more robust and methodologically comparable studies.

Several mechanisms may explain the observed effects. *H. coagulans* may inhibit cariogenic bacteria through competitive exclusion, production of antimicrobial peptides, and disruption of biofilm formation (50). In addition, it may exert anti-inflammatory effects by modulating cytokine production and oxidative stress markers, as evidenced by reductions in gingival bleeding and Glutathione Peroxidase activity in some trials (23). Unlike chemical antiseptics such as chlorhexidine, probiotics aim to restore microbial balance rather than indiscriminately suppress the oral microbiota, which could explain their favorable safety profile.

The evidence base is strengthened by the predominance of randomized controlled trials, the inclusion of both pediatric and adult populations, and the use of multiple delivery vehicles (tablets, mouthwash, food matrices). Nonetheless, several limitations must be acknowledged. The studies were highly heterogeneous in strain dosage, intervention duration, outcome measures, and control conditions, which precluded robust meta-analysis for most outcomes. Sample sizes were generally limited, and several studies exhibited an unclear or high risk of bias, primarily due to insufficient reporting of randomization procedures and the absence of blinding, which may have introduced methodological weaknesses. Moreover, follow-up periods were quite short, reducing insights into the durability of probiotic effects.

Despite these limitations, the findings may have relevant clinical implications. *H. coagulans* could serve as a safe adjunct to conventional caries prevention measures, particularly in children at high risk of caries development. Its use in mouthwash or tablets may offer practical advantages for daily application and better compliance. The observed improvements in gingival inflammation indicate potential benefits for individuals with mild periodontal disease, positioning *H. coagulans* as a useful adjunct to conventional mechanical plaque control and professional prophylaxis. Notably, no significant adverse effects were reported, further supporting its safety and potential suitability for long-term preventive applications.

Further well-designed randomized controlled trials are necessary to confirm these preliminary results and to address remaining gaps. Future studies should standardize probiotic dosages, use validated outcome measures, and include longer follow-up periods to assess the persistence of effects. Investigations employing next-generation sequencing could provide deeper insight into changes in the oral microbiome and clarify whether *H. coagulans* promotes a sustainable shift toward health-associated microbial communities. Trials evaluating caries incidence, periodontal disease progression, and patient-reported outcomes would help determine the true clinical significance of the observed microbiological changes.

## Conclusions

Overall, this systematic review indicates that current evidence is insufficient to support the clinical efficacy of *H. coagulans* in improving oral health. Nonetheless, the available findings are encouraging and suggest that this species may have potential as a future adjunct in oral care. High-quality, large-scale clinical trials are needed to determine optimal dosing, delivery methods, and long-term effectiveness, and to clarify the precise role of *H. coagulans* in preventive and therapeutic oral health strategies.
