# Supplementary material for: Oral health benefits of Heyndrickxia coagulans: a systematic review and meta-analysis of current evidence
Source: Front Oral Health. 2026 Jan 20;6:1733955. doi: 10.3389/froh.2025.1733955 (PMC12864477; doi:10.3389/froh.2025.1733955)
Supplement: Supplementary file 1 [file Table1.docx]

Supplementary Material

1S. Prisma checklist.

2S. Search strings.

3S. Customized data collection form used for data extraction from the included studies.

4S. Title and abstract screening records.

5S. Full text screening records.

6S. Risk of bias assessment of NRSI, performed with ROBINS-I.

1S. Prisma checklist.

| **Section and Topic** | **Item #** | **Checklist item** | **Location where item is reported** | |  |
| --- | --- | --- | --- | --- | --- |
| **TITLE** | | | |  | |
| Title | 1 | Identify the report as a systematic review. | 1 | |  |
| **ABSTRACT** | | | |  | |
| Abstract | 2 | See the PRISMA 2020 for Abstracts checklist. | 1 | |  |
| **INTRODUCTION** | | | |  | |
| Rationale | 3 | Describe the rationale for the review in the context of existing knowledge. | 1-2 | |  |
| Objectives | 4 | Provide an explicit statement of the objective(s) or question(s) the review addresses. | 2 | |  |
| **METHODS** | | | |  | |
| Eligibility criteria | 5 | Specify the inclusion and exclusion criteria for the review and how studies were grouped for the syntheses. | 3-4 | |  |
| Information sources | 6 | Specify all databases, registers, websites, organisations, reference lists and other sources searched or consulted to identify studies. Specify the date when each source was last searched or consulted. | 4 | |  |
| Search strategy | 7 | Present the full search strategies for all databases, registers and websites, including any filters and limits used. | 4 | |  |
| Selection process | 8 | Specify the methods used to decide whether a study met the inclusion criteria of the review, including how many reviewers screened each record and each report retrieved, whether they worked independently, and if applicable, details of automation tools used in the process. | 4 | |  |
| Data collection process | 9 | Specify the methods used to collect data from reports, including how many reviewers collected data from each report, whether they worked independently, any processes for obtaining or confirming data from study investigators, and if applicable, details of automation tools used in the process. | 4 | |  |
| Data items | 10a | List and define all outcomes for which data were sought. Specify whether all results that were compatible with each outcome domain in each study were sought (e.g. for all measures, time points, analyses), and if not, the methods used to decide which results to collect. | 4 | |  |
|  | 10b | List and define all other variables for which data were sought (e.g. participant and intervention characteristics, funding sources). Describe any assumptions made about any missing or unclear information. | 4 | |  |
| Study risk of bias assessment | 11 | Specify the methods used to assess risk of bias in the included studies, including details of the tool(s) used, how many reviewers assessed each study and whether they worked independently, and if applicable, details of automation tools used in the process. | 4 | |  |
| Effect measures | 12 | Specify for each outcome the effect measure(s) (e.g. risk ratio, mean difference) used in the synthesis or presentation of results. | 4-5 | |  |
| Synthesis methods | 13a | Describe the processes used to decide which studies were eligible for each synthesis (e.g. tabulating the study intervention characteristics and comparing against the planned groups for each synthesis (item #5)). | 4-5 | |  |
|  | 13b | Describe any methods required to prepare the data for presentation or synthesis, such as handling of missing summary statistics, or data conversions. | 4-5 | |  |
|  | 13c | Describe any methods used to tabulate or visually display results of individual studies and syntheses. | 4-5 | |  |
|  | 13d | Describe any methods used to synthesize results and provide a rationale for the choice(s). If meta-analysis was performed, describe the model(s), method(s) to identify the presence and extent of statistical heterogeneity, and software package(s) used. | 4-5 | |  |
|  | 13e | Describe any methods used to explore possible causes of heterogeneity among study results (e.g. subgroup analysis, meta-regression). | 4-5 | |  |
|  | 13f | Describe any sensitivity analyses conducted to assess robustness of the synthesized results. | 4-5 | |  |
| Reporting bias assessment | 14 | Describe any methods used to assess risk of bias due to missing results in a synthesis (arising from reporting biases). | 4 | |  |
| Certainty assessment | 15 | Describe any methods used to assess certainty (or confidence) in the body of evidence for an outcome. | 4-5 | |  |
| **RESULTS** | | | |  | |
| Study selection | 16a | Describe the results of the search and selection process, from the number of records identified in the search to the number of studies included in the review, ideally using a flow diagram. | 5 | |  |
|  | 16b | Cite studies that might appear to meet the inclusion criteria, but which were excluded, and explain why they were excluded. | 5 | |  |
| Study characteristics | 17 | Cite each included study and present its characteristics. | 5 | |  |
| Risk of bias in studies | 18 | Present assessments of risk of bias for each included study. | 7 | |  |
| Results of individual studies | 19 | For all outcomes, present, for each study: (a) summary statistics for each group (where appropriate) and (b) an effect estimate and its precision (e.g. confidence/credible interval), ideally using structured tables or plots. | 5-6 | |  |
| Results of syntheses | 20a | For each synthesis, briefly summarise the characteristics and risk of bias among contributing studies. | 5-6-7 | |  |
|  | 20b | Present results of all statistical syntheses conducted. If meta-analysis was done, present for each the summary estimate and its precision (e.g. confidence/credible interval) and measures of statistical heterogeneity. If comparing groups, describe the direction of the effect. | 5-6-7 | |  |
|  | 20c | Present results of all investigations of possible causes of heterogeneity among study results. | 5-6-7 | |  |
|  | 20d | Present results of all sensitivity analyses conducted to assess the robustness of the synthesized results. | 5-6-7 | |  |
| Reporting biases | 21 | Present assessments of risk of bias due to missing results (arising from reporting biases) for each synthesis assessed. | 7 | |  |
| Certainty of evidence | 22 | Present assessments of certainty (or confidence) in the body of evidence for each outcome assessed. | 7 | |  |
| **DISCUSSION** | | | |  | |
| Discussion | 23a | Provide a general interpretation of the results in the context of other evidence. | 7-8-9 | |  |
|  | 23b | Discuss any limitations of the evidence included in the review. | 8-9 | |  |
|  | 23c | Discuss any limitations of the review processes used. | 8-9 | |  |
|  | 23d | Discuss implications of the results for practice, policy, and future research. | 9 | |  |
| **OTHER INFORMATION** | | | |  | |
| Registration and protocol | 24a | Provide registration information for the review, including register name and registration number, or state that the review was not registered. | 7 | |  |
|  | 24b | Indicate where the review protocol can be accessed, or state that a protocol was not prepared. | 7 | |  |
|  | 24c | Describe and explain any amendments to information provided at registration or in the protocol. | 7 | |  |
| Support | 25 | Describe sources of financial or non-financial support for the review, and the role of the funders or sponsors in the review. | 14 | |  |
| Competing interests | 26 | Declare any competing interests of review authors. | 14 | |  |
| Availability of data, code and other materials | 27 | Report which of the following are publicly available and where they can be found: template data collection forms; data extracted from included studies; data used for all analyses; analytic code; any other materials used in the review. | 14 | |  |

2S. Search strings.

| **Database** | **String** |
| --- | --- |
| Pubmed | ("Bacillus coagulans"[MeSH Terms] OR "Bacillus coagulans"[All Fields] OR "Weizmannia coagulans"[All Fields] OR "Heyndrickxia coagulans"[All Fields] OR [“coagulans"[Title/Abstract]](https://pubmed.ncbi.nlm.nih.gov/?term=%22hip+pain%22%5BTitle%2FAbstract%3A%7E2%5D)) AND ("probiotics"[MeSH Terms] OR "probiotic*"[All Fields]) AND (“dent*”[All Fields] OR “gingivitis”[All Fields] OR “periodontitis”[All Fields] OR “dental caries”[All Fields] OR “tooth decay”[All Fields] OR “mutans streptococc*”[All Fields] OR “streptococcus mutans”[All Fields] OR “oral health”[All Fields] OR “oral cavity”[All Fields] |
| Scopus | TITLE-ABS-KEY ( "Bacillus coagulans" OR "Weizmannia coagulans" OR "Heyndrickxia coagulans" ) AND TITLE-ABS-KEY ("probiotic*" ) AND TITLE-ABS-KEY( “dent*”OR “gingivitis” OR “periodontitis” OR “dental caries” OR “tooth decay” OR “mutans streptococc*” OR “streptococcus mutans” OR “oral health” OR “oral cavity” ) |
| Embase | (‘Bacillus coagulans’/exp OR ‘Weizmannia coagulans’/exp OR ‘Heyndrickxia coagulans’/exp OR ‘coagulans’/exp) AND (‘probiotic*’/exp) AND (‘dent*’/exp OR ‘gingivitis’/exp OR ’periodontitis’/exp OR ’dental caries’/exp OR ’tooth decay’/exp OR ’mutans streptococc*’/exp OR ’streptococcus mutans’/exp OR ’oral health’/exp or ‘oral cavity’/exp) |

3S. Customized data collection form used for data extraction from the included studies.

| ID |  |
| --- | --- |
| Author |  |
| Years |  |
| Location |  |
| Journal |  |
| Population |  |
| Gender |  |
| Age means and/or range (year) |  |
| Sample test (number) |  |
| Sample control (n) |  |
| Probiotic intervention |  |
| Delivery |  |
| Amount |  |
| Day of administration |  |
| Placebo/other therapy/control |  |
| Outcomes |  |
| Baseline test (mean) |  |
| Baseline test (SD) |  |
| Follow up test (mean) |  |
| Follow up test (SD) |  |
| Baseline control (mean) |  |
| Baseline control (SD) |  |
| Follow up control (mean) |  |
| Follow up t control (SD) |  |
| p-Value intra-group test |  |
| p-Value intra-group control |  |
| p-Value inter-group |  |

4S. Title and abstract screening records.

| **ID** | **Authors** | **Title** | **Journal / year** | **Doi or URL or Protocol registration** | **Reviewer 1** | **Reviewer 2** |
| --- | --- | --- | --- | --- | --- | --- |
| 1 | A, A.J. and Suresh, A. | Oral microbial shift induced by probiotic Bacillus coagualans along with its clinical perspectives | Journal of Oral Biology and Craniofacial Research / 2023 | 10.1016/j.jobcr.2023.03.013 | Excluded | Excluded |
| 2 | Addae, H.Y. and Apprey, C. and Kwarteng, A. | Gut Microbiome-Targeted Nutrition Interventions and Growth among Children in Low- and Middle-Income Countries: A Systematic Review and Meta-Analysis | Curr. Dev. Nutr. / 2024 | 10.1016/j.cdnut.2024.102085 | Excluded | Excluded |
| 3 | Banas, J.A. and Popp, E.T. | Recovery of Viable Bacteria from Probiotic Products that Target Oral Health | Probiotics and Antimicrobial Proteins / 2013 | 10.1007/s12602-013-9142-2 | Excluded | Excluded |
| 4 | Bhagwat, V.G. and Tattimani, S.V.G. and Baig, M.R. | Dietary Supplementation of Synbiotic Formulation with Phytoactives on Broiler Performance, Relative Ready-to-Cook Weight, Health, Nutrient Digestibility, Gut Health, and Litter Characteristics | J. Appl. Biol. Biotechnol. / 2023 | 10.7324/JABB.2023.11515 | Excluded | Excluded |
| 5 | Bogdanović, M. and Mladenović, D. and Mojovic, L. and Djuris, J. and Djukić-Vuković, A. | Intraoral administration of probiotics and postbiotics: An overview of microorganisms and formulation strategies | Brazilian Journal of Pharmaceutical Sciences / 2024 | 10.1590/s2175-97902024e23272 | Excluded | Excluded |
| 6 | Bungau, S.G. and Behl, T. and Singh, A. and Sehgal, A. and Singh, S. and Chigurupati, S. and Vijayabalan, S. and Das, S. and Palanimuthu, V.R. | Targeting probiotics in rheumatoid arthritis | Nutrients / 2021 | 10.3390/nu13103376 | Excluded | Excluded |
| 7 | Burezq, H. | Feed Additives and their Multiple Beneficial Effects in Sheep Production and Health | Indian Vet. J. / 2022 | https://www.researchgate.net/publication/363730948_Feed_Additives_and_their_Multiple_Beneficial_Effects_in_Sheep_Production_and_Health | Excluded | Excluded |
| 8 | Cho, M.-Y. and Eom, J.-H. and Choi, E.-M. and Yang, S.-J. and Lee, D. and Kim, Y.Y. and Kim, H.-S. and Hwang, I. | Recent advances in therapeutic probiotics: insights from human trials | Clinical Microbiology Reviews / 2025 | 10.1128/cmr.00240-24 | Excluded | Excluded |
| 9 | Cirio, S. and Salerno, C. and Guglielmetti, S.D. and Mezzasalma, V. and Sarrica, A. and Kirika, N. and Campus, G. and Cagetti, M.G. | In Vivo Study on the Salivary Kinetics of Two Probiotic Strains Delivered via Chewing Gum | Microorganisms / 2025 | 10.3390/microorganisms13040721 | Excluded | Excluded |
| 10 | Elghandour, M.M.M.Y. and Pacheco, E.B.F. and Khusro, A. and Tirado-González, D.N. and Lackner, M. and Ponce-Covarrubias, J.L. and De Palo, P. and Maggiolino, A. and Salem, A.Z.M. | Deciphering the role of Moringa oleifera seeds and probiotic bacteria on mitigation of biogas production from ruminants | AMB Express / 2024 | 10.1186/s13568-024-01744-x | Excluded | Excluded |
| 11 | Fu, C. and Shah, A.A. and Khan, R.U. and Khan, M.S. and Wanapat, M. | Emerging trends and applications in health-boosting microorganisms-specific strains for enhancing animal health | Microb. Pathog. / 2023 | 10.1016/j.micpath.2023.106290 | Excluded | Excluded |
| 12 | Ghuge, S. and Rahman, Z. and Bhale, N.A. and Dikundwar, A.G. and Dandekar, M.P. | Multistrain probiotic rescinds quinpirole-induced obsessive-compulsive disorder phenotypes by reshaping of microbiota gut-brain axis in rats | Pharmacol. Biochem. Behav. / 2023 | 10.1016/j.pbb.2023.173652 | Excluded | Excluded |
| 13 | Izadi, B. and Mohebbi-Fani, M. and Hosseinzadeh, S. and Shekarforoush, S.S. and Nazifi, S. and Rasooli, A. | Alteration of fatty acid profile of milk in Holstein cows fed Bacillus coagulans as probiotic: A field study | Iran. J. Vet. Res. / 2021 | 10.22099/IJVR.2021.38159.5558 | Excluded | Excluded |
| 14 | Jagadeesh, K.M. and Shenoy, N. and Talwar, A. and Shetty, S. | Clinical effect of pro-biotic containing Bacillus coagulans on plaque induced gingivitis: A randomised clinical pilot study | Nitte Univ. J. Health Sci. / 2017 | https://www.researchgate.net/publication/340858543_Clinical_effect_of_pro-biotic_containing_Bacillus_coagulans_on_plaque_induced_gingivitis_A_randomised_clinical_pilot_study | Included | Included |
| 15 | Ji, M. and Rong, X. and Wu, Y. and Li, H. and Zhao, X. and Zhao, Y. and Guo, X. and Cao, G. and Yang, Y. and Li, B. | Effects of Fermented Liquid Feed with Compound Probiotics on Growth Performance, Meat Quality, and Fecal Microbiota of Growing Pigs | Animals / 2025 | 10.3390/ani15050733 | Excluded | Excluded |
| 16 | Jindal, G. and Pandey, R.K. and Agarwal, J. and Singh, M. | A comparative evaluation of probiotics on salivary mutans streptococci counts in Indian children | European archives of paediatric dentistry : official journal of the European Academy of Paediatric Dentistry / 2011 | 10.1007/BF03262809 | Included | Included |
| 17 | Joerger, R.D. and Ganguly, A. | Current Status of the Preharvest Application of Proand Prebiotics to Farm Animals to Enhance the Microbial Safety of Animal Products | Microbiol. Spectr. / 2017 | 10.1128/microbiolspec.PFS-0012-2016 | Excluded | Excluded |
| 18 | Juárez-Chairez, M.F. and Cid-Gallegos, M.S. and Cristian, C. and Prieto-Contreras, L.F. and Bollain-Y-Goytia, J.J. | The role of microbiota on rheumatoid arthritis onset | International Journal of Rheumatic Diseases / 2024 | 10.1111/1756-185X.15122 | Excluded | Excluded |
| 19 | Jurenka, J.S. | Bacillus coagulans | Alternative Medicine Review / 2012 | https://pubmed.ncbi.nlm.nih.gov/22502625/ | Excluded | Excluded |
| 20 | Khalesi, S. and Bellissimo, N. and Vandelanotte, C. and Williams, S. and Stanley, D. and Irwin, C. | A review of probiotic supplementation in healthy adults: helpful or hype? | Eur. J. Clin. Nutr. / 2019 | 10.1038/s41430-018-0135-9 | Excluded | Excluded |
| 21 | Koopaie, M. and Fatahzadeh, M. and Jahangir, S. and Bakhtiari, R. | Comparison of the effect of regular and probiotic cake (Bacillus coagulans) on salivary ph and streptococcus mutans count | Dental and Medical Problems / 2019 | 10.17219/dmp/99757 | Included | Included |
| 22 | Koopaie, M. and Jahangir, S. and Bakhtiari, R. | Evaluation of the effect of short-term consumption of probiotic (Bacillus coagulans) and ordinary cake on salivary Streptococcus mutans: A pilot study | Journal of Babol University of Medical Sciences / 2018 | 10.18869/acadpub.jbums.20.9.48 | Included | Included |
| 23 | Krupa, N.C. and Thippeswamy, H.M. and Chandra Shekar, B.R. | Antimicrobial efficacy of Xylitol, Probiotic and Chlorhexidine mouth rinses among children and elderly population at high risk for dental caries - A Randomized Controlled Trial | Journal of Preventive Medicine and Hygiene / 2022 | 10.15167/2421-4248/jpmh2022.63.2.1772 | Included | Included |
| 24 | Kuo, C.-L. and Hsin-Hsien Yeh, S. and Chang, T.-M. and I-Chin Wei, A. and Chen, W.-J. and Chu, H.-F. and Tseng, A.-L. and Lin, P.-L. and Lin, Z.-C. and Peng, K.-T. and Liu, J.-F. | Bacillus coagulans BACO-17 ameliorates in vitro and in vivo progression of Rheumatoid arthritis | Int. Immunopharmacol. / 2024 | 10.1016/j.intimp.2024.112863 | Excluded | Excluded |
| 25 | Lambo, M.T. and Chang, X. and Liu, D. | The recent trend in the use of multistrain probiotics in livestock production: An overview | Animals / 2021 | 10.3390/ani11102805 | Excluded | Excluded |
| 26 | Lima-Engelmann, K. and Schneider, M. | Probiotic Formulation Development and Local Application with Focus on Local Buccal, Nasal and Pulmonary Application | Curr. Nutraceutical. / 2022 | 10.2174/2665978604666221122112434 | Excluded | Excluded |
| 27 | Malmir, H. and Ejtahed, H.-S. and Soroush, A.-R. and Mortazavian, A.M. and Fahimfar, N. and Ostovar, A. and Esmaillzadeh, A. and Larijani, B. and Hasani-Ranjbar, S. | Probiotics as a New Regulator for Bone Health: A Systematic Review and Meta-Analysis | Evid.-Based Complement. Altern. Med. / 2021 | 10.1155/2021/3582989 | Excluded | Excluded |
| 28 | Mansoor, A. and Mansoor, E. and Mehmood, M. and Hassan, S.M.U. and Shah, A.U. and Asjid, U. and Ishtiaq, M. and Jamal, A. and Rai, A. and Palma, P.J. | Novel microbial synthesis of titania nanoparticles using probiotic Bacillus coagulans and its role in enhancing the microhardness of glass ionomer restorative materials | Odontology / the Society of the Nippon Dental University / 2024 | 10.1007/s10266-024-00921-5 | Excluded | Excluded |
| 29 | Mazhar, S. and Simon, A. and Khokhlova, E. and Colom, J. and Leeuwendaal, N. and Deaton, J. and Rea, K. | In vitro safety and functional characterization of the novel Bacillus coagulans strain CGI314 | Frontiers in Microbiology / 2023 | 10.3389/fmicb.2023.1302480 | Excluded | Excluded |
| 30 | McFarlin BK, Deemer SE, Bridgeman EA. | Oral Spore-Based Probiotic Supplementation Alters Post-Prandial Expression of mRNA Associated with Gastrointestinal Health | Biomedicines / 2024 | 10.3390/biomedicines12102386 | Excluded | Excluded |
| 31 | McFarlin BK, Henning AL, Bowman EM, Gary MA, Carbajal KM. | Oral spore-based probiotic supplementation was associated with reduced incidence of post-prandial dietary endotoxin, triglycerides, and disease risk biomarkers | World J Gastrointest Pathophysiol. / 2017 | 10.4291/wjgp.v8.i3.117 | Excluded | Excluded |
| 32 | McFarlin, B.K. and Henning, A.L. and Bowman, E.M. and Gary, M.M. | Reversing meal-associated gastrointestinal gut permeability issues: Potential treatment target for spore-based probiotics? | Am. J. Gastroenterol. / 2017 | 10.1038/ajg.2017.309 | Excluded | Excluded |
| 33 | McFarlin, B.K. and Tanner, E.A. and Hill, D.W. and Vingren, J.L. | Prebiotic/probiotic supplementation resulted in reduced visceral fat and mRNA expression associated with adipose tissue inflammation, systemic inflammation, and chronic disease risk | Genes Nutr. / 2022 | 10.1186/s12263-022-00718-7 | Excluded | Excluded |
| 34 | Mitic, K. and Kaftandzieva, A. and Popovska, M. and Ivanovski, K. and Pandilova, M. and Georgieva, S. and Pesevska, S. and Atanasovska-Stojanovska, A. and Kapusevska, B. and Janev, E. and Mijovska, A. | Probiotics and oral health | Res. J. Pharm., Biol. Chem. Sci. / 2017 | https://www.embase.com/search/results?subaction=viewrecord&id=L616287824&from=export U2 - L616287824 | Included | Included |
| 35 | Miyamoto, H. and Seta, M. and Horiuchi, S. and Iwasawa, Y. and Naito, T. and Nishida, A. and Miyamoto, H. and Matsushita, T. and Itoh, K. and Kodama, H. | Potential probiotic thermophiles isolated from mice after compost ingestion | J. Appl. Microbiol. / 2013 | 10.1111/jam.12131 | Excluded | Excluded |
| 36 | Mohseni, A.H. and Casolaro, V. and Bermúdez-Humarán, L.G. and Keyvani, H. and Taghinezhad-S, S. | Modulation of the PI3K/Akt/mTOR signaling pathway by probiotics as a fruitful target for orchestrating the immune response | Gut Microbes / 2021 | 10.1080/19490976.2021.1886844 | Excluded | Excluded |
| 37 | Mu, Y. and Cong, Y. | Bacillus coagulans and its applications in medicine | Beneficial Microbes / 2019 | 10.3920/BM2019.0016 | Excluded | Excluded |
| 38 | NA | Gut Health and the Effect on Substance and Alcohol Cravings | clinicaltrials.gov / 2023 | Registration Number NCT06026982 | Excluded | Excluded |
| 39 | NA | Evaluation of the Effects of Probiotic Toothpastes on Periodontal Health | clinicaltrials.gov / 2024 | Registration Number NCT06514664 | Included | Included |
| 40 | NA | Evaluation of the Consumption of Probiotics on the Bacteria Causing Dental Caries: A Randomised Clinical Trial | clinicaltrials.gov / 2016 | Registration Number NCT02752594 | Included | Included |
| 41 | Nadali, N. and Pahlevanloo, A. and Sarabi, M. and Zomorodi, S. | Production of probiotic powdered barberry (Berberis vulgaris) juice by cast-tape drying technique | LWT / 2023 | 10.1016/j.lwt.2023.115513 | Excluded | Excluded |
| 42 | Noman, M. and Kazmi, S.S.U.H. and Saqib, H.S.A. and Fiaz, U. and Pastorino, P. and Barcelò, D. and Tayyab, M. and Liu, W. and Wang, Z. and Yaseen, Z.M. | Harnessing probiotics and prebiotics as eco-friendly solution for cleaner shrimp aquaculture production: A state of the art scientific consensus | Sci. Total Environ. / 2024 | 10.1016/j.scitotenv.2024.169921 | Excluded | Excluded |
| 43 | Patel, S. and Patel, M. and Kaushik, G. and Patel, M.S. and Desai, J. and Patel, M. | Innovative Fusion of Probiotics and Mouth Fresheners: Investigating Unaltered Growth, Microscopic Analysis, and Mucoadhesive Strength | Int J Pharm Compd / 2024 | https://pubmed.ncbi.nlm.nih.gov/38768505/ | Excluded | Excluded |
| 44 | Rahmannia, M. and Poudineh, M. and Mirzaei, R. and Aalipour, M.A. and Shahidi Bonjar, A.H. and Goudarzi, M. and Kheradmand, A. and Aslani, H.R. and Sadeghian, M. and Nasiri, M.J. and Sechi, L.A. | Strain-specific effects of probiotics on depression and anxiety: a meta-analysis | Gut Pathogens / 2024 | 10.1186/s13099-024-00634-8 | Excluded | Excluded |
| 45 | Ratna Sudha, M. and Neelamraju, J. and Surendra Reddy, M. and Kumar, M. | Evaluation of the Effect of Probiotic Bacillus coagulans Unique IS2 on Mutans Streptococci and Lactobacilli Levels in Saliva and Plaque: A Double-Blind, Randomized, Placebo-Controlled Study in Children | International Journal of Dentistry / 2020 | 10.1155/2020/8891708 | Included | Included |
| 46 | Roy, A. and Kumar, Y. and Fatima, S. | A prospective, randomized, single-center, two-arm, open-label study to evaluate the efficacy of biotherapi ®, a two-strain bacillus probiotic blend, as an adjunctive therapy in the treatment of rheumatoid arthritis | Indian J. Rheumatol. / 2021 | 10.4103/injr.injr_281_20 | Excluded | Excluded |
| 47 | Rupp, S.K. and Stengel, A. | Bi-Directionality of the Microbiota-Gut-Brain Axis in Patients With Functional Dyspepsia: Relevance of Psychotherapy and Probiotics | Front. Neurosci. / 2022 | 10.3389/fnins.2022.844564 | Excluded | Excluded |
| 48 | Siezen, R.J. and Wilson, G. | Probiotics genomics | Microbial Biotechnology / 2010 | 10.1111/j.1751-7915.2009.00159.x | Excluded | Excluded |
| 49 | Sivri, D. and Şeref, B. and Şare Bulut, M. and Gezmen-Karadaǧ, M. | Evaluation of the Effect of Probiotic Supplementation on Intestinal Barrier Integrity and Epithelial Damage in Colitis Disease: A Systematic Review | Nutrition Reviews / 2025 | 10.1093/nutrit/nuae180 | Excluded | Excluded |
| 50 | Spaggiari L, Ardizzoni A, Pedretti N, Iseppi R, Sabia C, Russo R, Kenno S, De Seta F, Pericolini E. | Bacillus coagulans LMG S-24828 Impairs Candida Virulence and Protects Vaginal Epithelial Cells against Candida Infection In Vitro | Microorganisms / 2024 | 10.3390/microorganisms12081634 | Excluded | Excluded |
| 51 | Srividya, A.R. and Vishnuvarthan, V.J. | Probiotic: A rational approach to use probiotic as medicine | Int. J. Pharm. Front. Res. / 2011 | NA | Excluded | Excluded |
| 52 | Verma, A. and Inslicht, S.S. and Bhargava, A. | Gut-Brain Axis: Role of Microbiome, Metabolomics, Hormones, and Stress in Mental Health Disorders | Cells / 2024 | 10.3390/cells13171436 | Excluded | Excluded |
| 53 | Wallace, C. and Gordon, M. and Sinopoulou, V. and Akobeng, A.K. | Probiotics for management of functional abdominal pain disorders in children | Cochrane Database Syst. Rev. / 2023 | 10.1002/14651858.CD012849.pub2 | Excluded | Excluded |
| 54 | Xie, P. and Luo, M. and Deng, X. and Fan, J. and Xiong, L. | Outcome-Specific Efficacy of Different Probiotic Strains and Mixtures in Irritable Bowel Syndrome: A Systematic Review and Network Meta-Analysis | Nutrients / 2023 | 10.3390/nu15173856 | Excluded | Excluded |
| 55 | Yendluru MS, Manne RK, Kannan N, Bepari AS, Anumula A, Pulimi S. | Probiotics an Adjuvant in The Management of Recurrent Aphthous Ulcer: A Randomized Clinical Trial | J Indian Acad Oral Med Radiol / 2020 | 10.4103/jiaomr.jiaomr_47_20 | Included | Included |

Table 5S. Full text screening records.

| **ID** | **Authors** | **Title** | **Journal / year** | **Doi or URL or Protocol registration** | **Reviewer 1** | **Reviewer 2** | **Reason for exclusion** |
| --- | --- | --- | --- | --- | --- | --- | --- |
| 14 | Jagadeesh, K.M. and Shenoy, N. and Talwar, A. and Shetty, S. | Clinical effect of pro-biotic containing Bacillus coagulans on plaque induced gingivitis: A randomised clinical pilot study | Nitte Univ. J. Health Sci. / 2017 | https://www.embase.com/search/results?subaction=viewrecord&id=L620487328&from=export U2 - L620487328 | Included | Included |  |
| 16 | Jindal, G. and Pandey, R.K. and Agarwal, J. and Singh, M. | A comparative evaluation of probiotics on salivary mutans streptococci counts in Indian children | European archives of paediatric dentistry : official journal of the European Academy of Paediatric Dentistry / 2011 | 10.1007/BF03262809 | Included | Included |  |
| 21 | Koopaie, M. and Fatahzadeh, M. and Jahangir, S. and Bakhtiari, R. | Comparison of the effect of regular and probiotic cake (Bacillus coagulans) on salivary ph and streptococcus mutans count | Dental and Medical Problems / 2019 | 10.17219/dmp/99757 | Included | Included |  |
| 22 | Koopaie, M. and Jahangir, S. and Bakhtiari, R. | Evaluation of the effect of short-term consumption of probiotic (Bacillus coagulans) and ordinary cake on salivary Streptococcus mutans: A pilot study | Journal of Babol University of Medical Sciences / 2018 | 10.18869/acadpub.jbums.20.9.48 | Included | Included |  |
| 23 | Krupa, N.C. and Thippeswamy, H.M. and Chandra Shekar, B.R. | Antimicrobial efficacy of Xylitol, Probiotic and Chlorhexidine mouth rinses among children and elderly population at high risk for dental caries - A Randomized Controlled Trial | Journal of Preventive Medicine and Hygiene / 2022 | 10.15167/2421-4248/jpmh2022.63.2.1772 | Included | Included |  |
| 34 | Mitic, K. and Kaftandzieva, A. and Popovska, M. and Ivanovski, K. and Pandilova, M. and Georgieva, S. and Pesevska, S. and Atanasovska-Stojanovska, A. and Kapusevska, B. and Janev, E. and Mijovska, A. | Probiotics and oral health | Res. J. Pharm., Biol. Chem. Sci. / 2017 | https://www.embase.com/search/results?subaction=viewrecord&id=L616287824&from=export U2 - L616287824 | Included | Included |  |
| 39 | NA | Evaluation of the Effects of Probiotic Toothpastes on Periodontal Health | clinicaltrials.gov / 2024 | Registration Number NCT06514664 | Excluded | Excluded | Registration of study protocol |
| 40 | NA | Evaluation of the Consumption of Probiotics on the Bacteria Causing Dental Caries: A Randomised Clinical Trial | clinicaltrials.gov / 2016 | Registration Number NCT02752594 | Excluded | Excluded | Registration of study protocol |
| 45 | Ratna Sudha, M. and Neelamraju, J. and Surendra Reddy, M. and Kumar, M. | Evaluation of the Effect of Probiotic Bacillus coagulans Unique IS2 on Mutans Streptococci and Lactobacilli Levels in Saliva and Plaque: A Double-Blind, Randomized, Placebo-Controlled Study in Children | International Journal of Dentistry / 2020 | 10.1155/2020/8891708 | Included | Included |  |
| 50 | Yendluru MS, Manne RK, Kannan N, Bepari AS, Anumula A, Pulimi S. | Probiotics an Adjuvant in The Management of Recurrent Aphthous Ulcer: A Randomized Clinical Trial | J Indian Acad Oral Med Radiol / 2020 | 10.4103/jiaomr.jiaomr_47_20 | Included | Included |  |

6S. Risk of bias assessment of NRSI, performed with ROBINS-I.

| Authors, year | Experimental | | | Comparator | Outcome |
| --- | --- | --- | --- | --- | --- |
| **Mitic et al. 2017** | Scaling and Root Planing *(SRP) + H. coagulans*, *L. acidophilus, S. thermophilus, L. bulgaricus, B. bifidum* | | | Scaling and Root Planing (SRP) | Probing Depth (PD) |
| **Domain** | | **Judgment** |  | **Comment** | |
| Bias due to confounding | | Moderate | 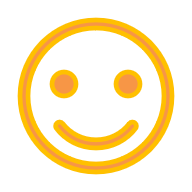 | The study excluded smokers and patients with systemic diseases, but did not use multivariate analyses to control for other confounders. | |
| Bias in selection of participants into the study | | Low | 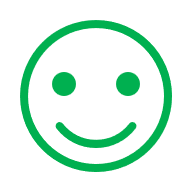 | Clear and enforced inclusion/exclusion criteria prior to surgery. | |
| Bias in classification of interventions | | Low | 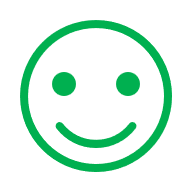 | Well-defined intervention with standardized composition, dosage and duration. | |
| Bias due to deviations from intended interventions | | Moderate | 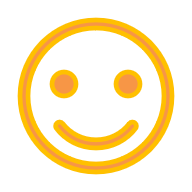 | No compliance monitoring and no blinding. | |
| Bias due to missing data | | Low | 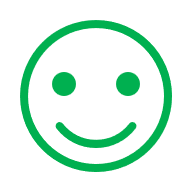 | No drop-outs were reported and all patients completed the study. | |
| Bias in measurement of outcomes | | Moderate | 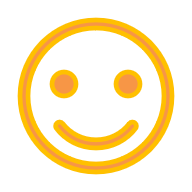 | Standardized clinical outcomes, but it is unclear whether the assessors were treatment-blind. | |
| Bias in selection of the reported result | | Moderate | 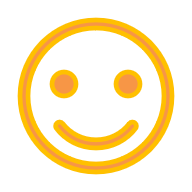 | Key results reported, but no pre-registered protocols mentioned. | |
| **Overall** | | **Moderate** | 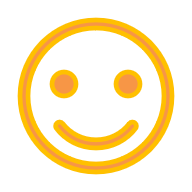 |  | |
